# Social media in the infertile community—using a text analysis tool to identify the topics of discussion on the multitude of infertility blogs

**DOI:** 10.1177/17455065211063280

**Published:** 2021-12-03

**Authors:** Taina Sormunen, Margareta Westerbotn, Arthur Aanesen, Bjöörn Fossum, Klas Karlgren

**Affiliations:** 1 Department of Clinical Science and Education, Karolinska Institutet, Södersjukhuset, Stockholm, Sweden; 2 Department of Health Promoting Science, Sophiahemmet University, Stockholm, Sweden; 3 Department of Nursing Science, Sophiahemmet University, Stockholm, Sweden; 4 Sophiahemmet Hospital, Stockholm, Sweden; 5 Department of Learning, Informatics, Management and Ethics, Karolinska Institutet, Stockholm, Sweden; 6 Department of Research, Education, Development, and Innovation, Södersjukhuset, Stockholm, Sweden; 7 Department of Health and Functioning, Faculty of Health and Social Sciences, Western Norway University of Applied Sciences, Bergen, Norway

**Keywords:** blog, content analysis, diary, Gavagai Explorer, infertility, Internet

## Abstract

**Background:**

Infertility affects one in six couples. New digital resources exist which enable the study of lived experience of persons with infertility. Blogging represents a forum for sharing narratives and experiences. To provide high quality care for persons with a history of infertility, it is crucial to ascertain what they value as significant in their situation. Blogs with a focus on infertility may provide this information.

**Objectives:**

The aim of this study was to gain insight into which infertility-related issues are discussed on Swedish infertility blogs.

**Methods:**

In total, 70 infertility blogs were identified on the Internet and 25 met the inclusion criteria. A quantitative–qualitative content analysis was performed with the support of the Gavagai Explorer text analysis software.

**Results:**

A total of 4508 postings were retrieved from the blogs, all of which were written by women. The outcome of the analysis resulted into the following topics: *Emotions* (16.8%), *Relations* (12.5%), *Time and waiting* (7.1%), *Body* (6.6%), *Care and treatment* (4.2%), *Food and diet* (1.4%) and *Exercise* (0.5%). For most topics, there was a balance between positive and negative statements, but the body topic stood out by having more negative than positive sentiment.

**Conclusion:**

By considering the topics expressed in blogs, health care personnel are provided with an opportunity to better understand the situation of individuals affected by infertility.

## Introduction

Infertility remains a continuous universal challenge.^1^ The clinical definition of infertility is “a disease of the reproductive system defined by the failure to achieve a clinical pregnancy after 12 months or more of regular unprotected sexual intercourse.”^2^ Globally, infertility is estimated to affect between 8% and 12% of reproductive-aged couples.^3^ Ovulation disorders, such as polycystic ovarian syndrome (PCOS) and amenorrhea, are the cause of infertility in approximately 25% of couples who have difficulties conceiving. These disorders are characterized by imbalances in the female hormones.^4^ Infertility in men can be caused by low numbers and/or poor functioning of sperm. Hormonal disorders may lead to low production of sperms, which may also contribute to difficulties in fertilization.^5^

Treatments for infertility are chosen based on the contributing infertility factor, the duration of infertility and the age of the female partner. Infertility can be treated with medicines, or in combination with intrauterine insemination (IUI) and assisted reproductive technologies (ART), such as in vitro fertilization (IVF).^6^ In Sweden, a maximum of three full IVF cycles leading to the couple’s first child are reimbursed by the Swedish general health care system.^7^ The experience of infertility can be distressing, unexpected and a life changing experience.^8^ The sense of loss can cause depression^9^ and even suicidal thoughts.^10^ Infertility generates a major emotional burden for the couples affected. Women seem to be more at risk to suffer from the psychological effects of infertility compared to men^11^ and women who are at the beginning of treatments may be more emotionally affected.^12^

Blogs can be considered as a development of diary writing^13^ and have been described as being a “protected space” allowing bloggers to share their narratives, with an invisible audience without interruption.^14^ Private blogs are password-protected and anonymous. However, the majority of open blogs are detectable and easily discovered by search engines because they are registered by the users’ blog service.^13^ In Sweden, 98% of the population have access to the Internet and 63% of the Internet users use social networks daily.^15^

Infertility, sometimes referred as the “silent disorder,” has received a voice through blogs and social media.^16^ The blogosphere provides infertile individuals with the opportunity to create a community, narrate their private stories, seek support and raise awareness about their disorder.^17^ Furthermore, previous studies have shown that women with infertility used blogs as a source of information and for social support.^18^ Women are more active in social media and infertility is one of the most frequently covered health-related topics.^19^ Online social media forums can provide information and support that may not be possible to obtain in the interaction with health care personnel^20,21^ and the forums may be particularly important to those who lack emotional support elsewhere.^22^ Findings of one previous study about communication among infertile women show that some women have difficulties to discuss infertility-related issues with their spouses.^23^ Health care personnel provide information to patients which is often based on their own assumptions of patients’ needs,^24^ but patients report that they may turn to online support groups to find information that is not provided by medical professionals.^25^ As scheduled, medical appointments typically are constrained in time; the possibilities to discuss the medical background of patients are limited.^26^ However, understanding patients’ perspectives and witnessing their narrative are crucial for gaining insight into their situation^27^ motivating for investigation of the online arenas that women with infertility are turning to.

Blog texts can provide insight into what persons affected by infertility value as significant regarding their life situation. Increased knowledge about the life situations of these persons can help to improve offered care.^28^ Swedish blogs dealing with infertility are, however, relatively unexplored and it remains unknown how the topic of infertility is discussed on the blogs. Analysis of the plentiful writings of women with infertility may contribute to the understanding of the emotional, physical and psychological aspects of infertility, which otherwise risk being missed. Studies on blogs may act as a complement to existing knowledge about how women are affected by infertility. The aim was, therefore, to gain insight into which infertility-related issues are discussed on Swedish infertility blogs. The focus of this study was explorative and has a particular focus on the blog writers’ perspective.

## Methods

### Design

A qualitative–quantitative analysis of data was performed, using the Gavagai Explorer software to analyze the contents of Swedish infertility blogs. The analytic process involves both quantitative and qualitative elements, which are iteratively integrated during the analysis. It does not include separate sources of data and should be distinguished from a conventional mixed-methods study.

### Sampling and data collection

In total, 70 Swedish infertility blogs were identified, through a website for a Swedish infertility interest group www.villhabarn.se (in English “I want children”), which consists of information, articles and blogs. This specific webpage was chosen as it provides a listing of open infertility blogs in Sweden. Criterion sampling, which involves selecting cases that meet defined criteria of importance, was used. Inclusion criteria for the blogs: open access blogs related to infertility, written in Swedish in a diary style format by a person affected by infertility and which has been maintained at least 3 years and has postings accessible through blog archives.

All 25 blogs that met the criteria were included. Data were collected from May to September 2017. The bloggers’ posts were retrieved 3 years retroactively from their archives (2014–2017). A 3-year limit was set to obtain as current texts as possible and written when conditions were comparable; for example, legislative changes regarding IVF treatments had taken place previously.^29^ All blog texts in each blog written by the blogger were included in the analysis. Postings written by the readers of the blogs were not included in the analysis, as we had a focus on the perspective of the infertile women and did not have a way of verifying the identity of those commenting on the blogs.

The respective bloggers maintained their blog over a broad span of time, from 3 to 18 years. The blogs were established between 1999 and 2015. The total amount of postings on the blogs (3-year period) was 4508, from 42 postings to 643 per blog (median 180 postings per year). In 15 blogs (60%), the number of readers or followers was not stated clearly. In 10 blogs, the number of readers was indicated and was ranged, in total, between 150 and 1.4 million.

### Analysis

An interactive quantitative–qualitative inductive content analysis and a sentiment analysis were performed using the Gavagai Explorer^30^ software, developed for qualitative analysis is of large sets of text data.^31–34^ Content analysis is the analysis of the contents of narrative data to identify major themes and patterns.^35^

Use of the software enables analysis of large texts but requires a high degree of interactivity from the analysts. It also enables the analysts to group together responses which refer to the same subject or “topic” as they are called in the software.^36^ The tool analyzes texts and helps the analysts by suggesting a list of topics based on meaning. The system uses measures of term specificity in language to assess how specific or general a topic is, and it also ranks the topics according to the prevalence in the texts.^36^ In addition, the tool lists the related terms; terms are the actual words (or sequences of words) from the data that the topic is composed of. Terms or topics found in the data can be ignored or merged to dig deeper into the dataset.^36^ For instance, consider a topic, such as *Health care personnel* built on associated terms, such as *nurse, doctor, midwife, physician* and *dentist*. While all these associated terms could be argued to belong to the topic of health care personnel, some of these (e.g. *dentist*) might be considered irrelevant for the aim of the study and therefore discarded in the next iteration. Moreover, if a term appears to be missing in a topic (e.g. *gynecologist*) it can easily be added to make the topic more precise. A sentiment analysis was performed to study the sentiments associated with the topics in the blogs. Sentiment analysis, also known as opinion mining, is the use of natural language processing to systematically identify and quantify affective information expressed in a text. The Gavagai tool uses a lexical approach based on a curated list of sentiment bearing terms to quantify basic sentiments for each topic. In the case of this study, two basic sentiments were studied, namely whether the bloggers were writing about a topic, positively (e.g. being hopeful or confident) or negatively (e.g. being sad, unable to cope).^36^

The analysis was performed according to the following steps:

1. After collecting the postings, the first author read and reread the blog texts several times to gain an understanding of, and familiarity with, the content.
2. To prepare the analysis for the tool, all blog texts were saved in a comma-separated values (CSV) file, containing one sentence per row in a total of 33,020 sentences.
3. The research team analyzed the texts using the Gavagai Explorer with an iterative approach on multiple occasions. After each iteration, the topics suggested by the tool and their associated terms were scrutinized and the texts underlying the topics were studied. The topics were then further refined by the first, second and last authors through merging closely related topics into one or through grouping topics to contain subtopics. Topics irrelevant to the aim were discarded. New terms were added to the topics while others were removed to sharpen the analysis.
4. After each iteration, the tool recalculated the frequencies of the topics and suggested new topics and associated terms for the revised topics.^36,37^ Steps 3–4 continued until a consensus was reached and until no further new topics related to the aim were discovered. The software enables analysis of very large amounts of texts which may be difficult to analyze manually. The main advantage is, however, that the tool contributes to rigor in the analysis; the tool suggests topics to analyze and thereby the risk of overlooking or neglecting aspects of the text decreases. While it is ultimately the researchers who choose what to include and exclude in the analysis, the researchers will constantly (each iteration) be reminded about the topic unless they either include it in the analysis or make an active choice to exclude it.

About 50 iterations were performed, continuously going back and forth between parts and wholes of the texts and the analysis, that is, on the one hand, sentences, paragraphs and blog posts and on the other hand, terms and topics. Initial analyses were performed by the first, second and last authors and preliminary analyses were discussed critically with the entire research group during several recurring meetings before continuing. Credibility was ensured by continuously reflecting on and discussing the research focus and methods. Researcher triangulation was applied to reflect on data from different perspectives. The authors have experience of qualitative, quantitative and mixed-methods studies and bring complementary perspectives to the analysis; T.S. is a registered nurse (RN), M.W. is a midwife, A.A. is an obstetrician-gynecologist, B.F. is a pediatric nurse and professor of nursing and K.K. is a researcher within human–machine interaction and medical education.

As the analysis was inductive, a specific theory was not used to deductively direct the analysis. However, the project was based on the theoretical conviction that thinking is not only a mental process^38^ but may be distributed and mediated through artifacts and technologies,^39,40^ such as blogs, which therefore can be valuable resources in the interpretation of human thinking and experience. Dependability has been ensured by continuously tracking and reporting discussions and decisions and by descriptions of the analytic procedure, as above.

### Ethical considerations

Approval was obtained from the regional Ethical Review Board of Stockholm (EPN 2016/5:1). The research has been conducted according to ethical principles of research and good research practice concerning humans, in accordance with the Helsinki Declaration^41^ and the ICN Code of Ethics.^42^ Informed consent was not needed as the blogs used in this study were already existing and published allowing open access and unrestricted reading.

## Results

All blogs were written by women (n = 25) and allowed two-way communication, that is, responses from the blog visitors. The length and frequency of postings varied largely, from a few sentences to up to four pages (Table 1).

**Table 1.** Characteristics of the blogs.

Attributes	Frequency (%)
Anonymity	
Anonymous	20 (80)
Author named	5 (20)
Accessibility of the blogger	
E-mail	14 (56)
Via blog	11 (44)
Labels created by the blogger	
IVF	18 (72)
Infertility	14 (56)
Adoption	5 (20)
Oocyte donation	4 (16)
Endometriosis	2 (8)
Longing for a child	2 (8)

IVF: in vitro fertilization.

### Content analysis

**Figure 1.**
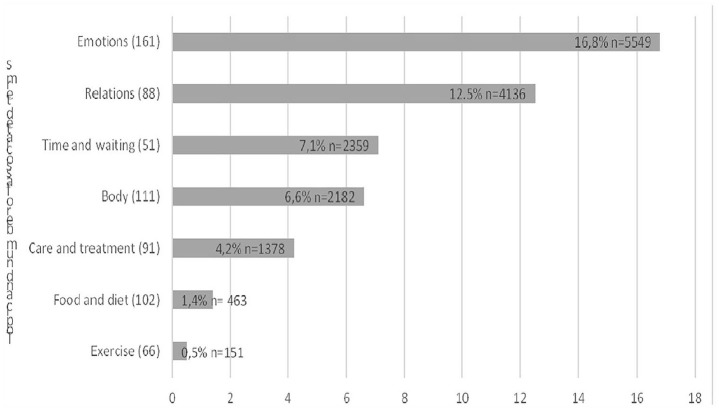
Topics related to infertility; (a total number of associated terms), percentage of occurrence out of 16,807 sentences for each topic and n = total number of sentences for each topic.

The content and depth of the blogs varied, some focusing on describing feelings and thoughts, others focusing on descriptions of everyday events. The blogs covered a large variety of issues and 16,807 of 33,020 (49.1%) sentences were related to infertility. The analysis showed that the bloggers frequently discussed infertility with reference to emotions, relations, time and waiting, body, care and treatment, food and diet and exercise (Figure 1).

#### Emotions

The most frequently occurring topic was *Emotions*, which consisted of 161 associated terms, identified in the texts, for example, worry, scared, stressed, relieved, grateful and enjoy. Being the largest topic, it also spanned many areas and was discussed in many different ways. While many postings were negative, many were also positive, and other discussed both positive and negative emotions. The bloggers described their feelings, ranging from extreme optimism to petrifying pessimism; “It’s hard to explain, but the feelings are like riding a roller coaster and I have no control.” The breadth and strength of emotions that women presented in relation to infertility were highs and lows, and not always predictable.

Feelings of fear and anxiety that the treatments would result in failure were evident in the blogs. The impact on daily life was obvious, with women describing an inability to conduct everyday activities because of thoughts about their infertility; “It is difficult, for some reason, to take control of a storm of emotions.”

Despite being busy and preoccupied with infertility, and with the pressures of everyday life, they occasionally took time to step back from their lives and appreciate the good things they had: “My life is filled with things I love and enjoy. I have a family, parents and siblings.” Seeing life in a wider context could help them put everyday worries in perspective and pay attention to the positive aspects of their day.

It emerged from the analysis that numerous women appreciated their relationship with their partner even more than before. In addition, appreciation of having friends, a rewarding job and opportunities to have holidays could help dispel thoughts about their infertility. They were grateful for having access to medical expertise and for fertility treatment provided by public health care. Successful treatment was described as the ultimate happiness.

#### Relations

Several of the bloggers wrote about their *Relations* and discussed family members, friends and described their appreciation of their spouses for providing support for them. This topic was composed of 88 associated terms, including partner, husband, friends, mother and virtual friend.

Several women wrote that they were unable to discuss thoughts or feelings about infertility with partners or friends and discussed how blogging replaced this need to a certain extent. They also asked for blog readers’ thoughts, reflections and opinions as described by a blogger: “Everyone does, of course, whatever feels right to them, but for me, it felt good to have people to talk to, someone who understands what it’s all about, someone who comes with encouraging words, support, comfort, and so on.”

Many of the bloggers described that new friendships were made through the infertility blogs. These friendships were described as valuable because they connected people in similar situations. Several bloggers actively sought feedback and advice from their blog readers and some also met followers offline.

The analysis of the blog texts revealed that the struggle with infertility affected spousal relationships negatively. The women blogged that their partners could not fully understand what the women were going through, such as irritability and other symptoms and demands of the fertility treatments. Due to stressful everyday life, they found it difficult to find time and energy for deeper discussions with their partners:

A moment ago, I was almost crying out of fatigue and was irritated with him. He is stressed about his job . . . I’ve had too much to do lately. We have not talked with each other properly for a long time and this leads to miscommunication.

#### Time and waiting

The *Time and Waiting* topic included 51 related terms identified in the texts, such as anticipation, hope, weeks, indefinite time, expectance and looking forward to the future and passing time with mixed feelings. On the one hand, these experiences were forward-looking and concerned hoping, anticipating and having faith in that all would go well. On the other hand, the postings covered frustration over having to wait and the associated feelings of anxiety, doubt and worry. Moreover, they also expressed relief in that time occasionally passed quickly. The blogs texts described how trying to conceive was a period of high anxiety, worry and frustration. The experience was described as straining—involving both positive and negative feelings at the same time: “And above all, I hope that confidence increases over the time, ahead of the next treatment cycle, sometime in the future . . . A good thing is that the summer is over soon, time passes, and the next cycle is approaching.”

#### Body

The *Body* topic was associated with 111 terms identified in the texts, for instance, failing body, pain, uterus and ovulation. This topic ranged between discussing a specific body part, overall and lasting bodily experiences, and their views of the body itself. They could, for instance, discuss an experience of pain in the stomach, how incessant fatigue affected them over time or how they viewed or related to their body. The women reported that infertility had led to negative feelings about their body. They felt betrayed by it: “If you, yourself are the cause of childlessness, it is sometimes hard to accept oneself, one’s own body.” Instead of doing what the body was “supposed to do,” it had disappointed them, and had failed in giving a child. Words, such as disgust and hate, were used. The women described in their texts experiencing physical pain related to fertility treatment, including side effects of medications (bloating, related to enlarging ovaries) and invasive procedures, such as egg retrieval, and they felt fed up.

#### Care and treatment

The topic *Care and treatment* consisted of 91 associated terms, including consultations, midwife contacts and making phone calls. This topic not only included postings which discussed planning and undergoing care and treatment but also the many related practical issues, such as planning and booking appointments, visiting clinics, maintaining contacts with the clinics and communicating with the personal. The bloggers described how the pursuit of fertility dominated every waking hour. They emphasized that they were experiencing an uncertain time with an outcome that was not clear. Practical items were described, such as how much time they spent making calls which was reported as time-consuming. Some women wrote that a lot of time was spent on traveling to the clinic and back. Few women wrote about the financial burden of infertility, although treatments can be costly.

Some women wrote about how they were greeted at the clinics, including experiences of both positive and negative reception from the staff: “The problem is that, even the thought of booking an appointment, going there and then having to talk about it (infertility) to a stranger again, feels like such a huge step which fills my eyes with tears, immediately.” Another woman described her experiences like this:

One more positive thing was that if we were to face the same problem again—no embryo to transfer—the doctor said they would like to give us a fourth try! It feels great that they could possibly give us another chance, when it has been the way it’s been.

Despite the many inconveniences regarding appointments and visits to the clinic, the bloggers tended to write in an appreciative manner about the encounters with the health care personnel and the blogs were not used for complaining about staff.

#### Food and diet

The topic *Food and diet* consisted of 102 related terms identified in the texts, for example, eating habits, weight, comfort eating, nourishment and unhealthy food. Food was described in terms of helping to reduce anxiety: “There is still another book to read on the couch, over a bowl of candy, there is no better relief.” Some bloggers wrote how they used to bake a cake for comfort food. Even lifestyle changes were made to decrease anxiety: Cooking wholesome meals was experienced by some of the bloggers as a great effort and time-consuming. Various diets were used to lose weight, such as replacement shakes and low carb diets: “Now I am focused on weight loss . . . it will be tough . . . but I am determined.”

#### Exercise

Finally, the *Exercise* topic included 66 associated terms, such as daily exercise, jogging, yoga and walks. The bloggers described how they participated in different activities, for example, walking in the woods and horseback riding. Emotions could be processed during solitary activities, and they allowed themselves to cry: “The training may not always be fun, but it definitely takes my mind off things and gives me a break.”

### Sentiment analysis

**Figure 2.**
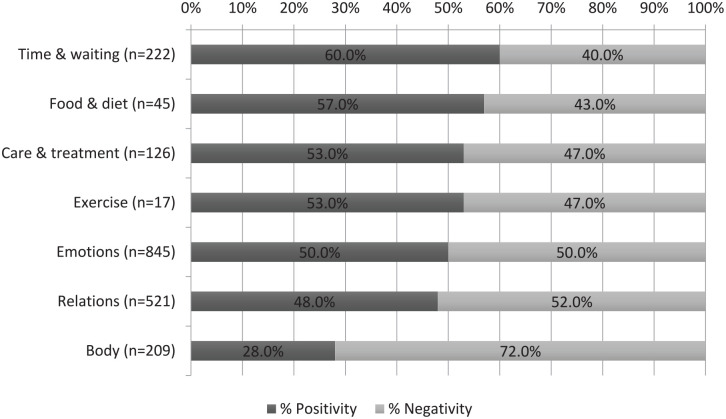
Sentiment analysis of sentences categorized either positive or negative: topic (number of included sentences) and percentage of the two sentiments.

As seen above, the blog topics could be quite polarized with both positive and negative texts. Results of the sentiment analysis (Figure 2) showed that the distribution of positive and negative sentiments were quite similar, except for the body topic, which to a higher degree was described negatively. Out of 209 sentences about the body, 150 (72%) were negative.

## Discussion

### Principal findings

The results show that *Emotions* was the most frequently occurring topic in the infertility blogs, followed by *Relations, Time and waiting* and *Body*. All included infertility blogs were written by female bloggers. Females are proportionally more active in generating blog content.^43^

Blog writing appeared to be a way of processing and working out issues and achieving emotional release.^44^ In line with previous research,^45^ the bloggers reported that the mental strain of infertility can be almost insufferable. The women processed their emotions through blogging. The experience of infertility is one of the greatest sources of life stress, and authoring a blog can help to reduce the psychological consequences.^46^ The results revealed that women used blogs for dealing with the emotional impact of infertility.

One of the most challenging aspects of advanced fertility treatment is dealing with the emotional, physical and social consequences of infertility. Although all women may share an experience, its manifestations are individual. Writing down what is on one’s mind in a blog can be a way to process one’s feelings and thoughts and to see things more clearly.^13^ The blogs not only describe the rough emotional fluctuations but also things that ease tough times, such as, one’s partner and activities.

The results indicated that partners may have a hard time understanding the situation these women are going through and therefore the women do not always want to discuss infertility-related issues with them. A previous study^23^ pointed out that women affected by infertility may have difficulties in discussing infertility-related issues with their partner. Through blogging they were able to meet, and discuss the situation with, other persons with a history of infertility. It may also be easier to write down feelings and thoughts than talk about them.^47^

The results revealed that waiting for test results and treatment could affect the women in a negative way. Previous research^48^ indicates that the experience of time is seemingly of fundamental importance for how persons make sense of the world. Time judgments are deeply emotional. The perception of time is linked to subjective well-being, and the passage of time differs depending on emotional stages.^49^ Regardless of which partner is the source of the fertility problem, it is the female partner who usually undergoes most of multi-step fertility treatment.^50^ Time management, such as keeping track of treatment timetables, may be of primary concern for them.

Fertility is a key function of the body^51^ and our results revealed that women felt betrayed by their body because it failed in giving them a child. The relation to one’s own body is existential, which means that the individual is her body.^52^ Infertility may mean an inability to participate in the world in a normal way and the women’s future desires in life may no longer be fulfilled. Their bodies inhibit their expectations and dreams of the future. Not only the emotional burden of infertility but also the physical pain caused by investigations and treatments, such as retrieval of oocytes, can be very stressful.^53^

Blogs with focus on infertility can provide authentic information about patients’ experiences and illness-related behavior.^14^ This important resource, that the patients themselves make available through their experiences and knowledge, cannot be ignored. By gaining more knowledge about how women experience infertility, health care personnel engaged in infertility treatment can better understand and pay attention to the situation and the needs of patients and thereby become better at providing person-centered care and support. The public nature of the Internet allows women affected by infertility to deal with this sensitive issue in their lives.^54^ Findings suggest that infertility blogs merge a mixture of issues and information, indicating that blogging serves as an outlet for these women. Understanding patients’ narratives can be helpful when planning care and to understand how patients react to care or treatment. In this study, we noted that while the bloggers described the trouble, they had related to booking appointments and visits to the clinic they remained appreciative rather than critical of the health care personnel. However, patients’ information needs are not always met during the short consultation time and therefore they are using the Internet to satisfy these needs. Current evidence shows that some women with fertility problems have difficulty discussing infertility-related issues with their spouse.^23^ Their postings about their frustration could perhaps be included in a critical discussion about the structure of care and we see the importance of having awareness of this arena for infertile women. This article contributes by providing an overview of women are expressing there, outside the meetings at the clinic.

### Strengths and limitations

A strength of the study is that all blogs and all their contents written between 2014 and 2017 were included in the analysis. The search strategy used focused solely on open blogs and excluded closed infertility blogs. A limitation is that not all individuals affected by infertility are equally likely to write about their medical experiences online. As a result, we may only view and analyze the experiences of a certain segment of the population.

The unsolicited narrative data from blogs are free from the influences of the research process itself, such as the risk of giving responses to please the interviewer.^55^ The anonymity afforded using pseudonyms may allow access to feelings and experiences more openly than may be the case in face-to-face interviews. Benefits comprise the ability to collect delicate information and to capture a variety of experiences.^56^ However, 80% of the blog authors in our study were anonymous. The “online mask” allows bloggers to write more freely and openly, which mitigates possible impression management.^57^ Fear of being hurt may be one of the reasons for blogging anonymously about infertility.^13^

The analysis of blogs, however, has limited opportunities to validate understanding or explore what is unsaid in the way that is possible in an interview situation.^58^ However, bloggers may share their thoughts and feelings in ways that they may not have done in an interview situation. Moreover, researchers can observe the lived experience of bloggers over prolonged periods of time without manipulating the process.^59^ A strength is that we have been able to provide an overview of the content of 4508 blog posts although it, for natural reasons, is not possible to present, in full detail, richness of all the data underlying each topic in the limited space of one article.

### Key message

In ever-growing digital society, new channels are created for dealing with adversities in life. All issues related to infertility cannot be raised during the brief appointment at the fertility clinic. Blogs are an important arena for infertile women to express and discuss questions that they have not found answers to elsewhere.

## Conclusion

Social media are used by women to process matters related to their infertility. Our study contributes to an understanding of which infertility-related aspects women ventilate in blogs beyond their contacts with health care personnel. Dominating topics include emotions, relations, time and waiting and treatment.
